# Amount of mechanical hip-knee-ankle (mHKA) angle correction by fixed-bearing medial UKA can be predicted using a new morphological assessment method: the arithmetic hip-knee-ankle (aHKA) angle

**DOI:** 10.1007/s00402-025-05892-y

**Published:** 2025-04-26

**Authors:** Naoki Nakano, Masanori Tsubosaka, Tomoyuki Kamenaga, Yuichi Kuroda, Kazunari Ishida, Shinya Hayashi, Ryosuke Kuroda, Tomoyuki Matsumoto

**Affiliations:** 1 Kobe University, Kobe, Japan; 2 Kobe Kaisei Hospital, Kobe, Japan

**Keywords:** Unicompartmental knee arthroplasty, Coronal alignment, Arithmetic hip-knee-ankle angle, Soft-tissue balance, Insert thickness

## Abstract

**Introduction:**

The amount of change in coronal alignment by unicompartmental knee arthroplasty (UKA) is important when considering its long-term results. This study investigated whether the amount of change in mechanical hip-knee-ankle (mHKA) angle by medial fixed-bearing UKA can be predicted based on the arithmetic hip-knee-ankle (aHKA) angle, an indicator of bony nature that is independent of soft tissue balance.

**Materials and methods:**

The research involved 101 patients (125 knees) who underwent medial fixed-bearing UKA with the spacer-block technique. Pre- and postoperative mHKA angles, aHKA angle (180°– lateral distal femoral angle + medial proximal tibial angle), insert thickness, and the amount of bone cuts were measured. The component gap in extension was measured using a UKA tensor, and the pre-osteotomy gap was calculated. The correlation between aHKA angle minus preoperative mHKA angle and changes in mHKA angle, as well as the pre-osteotomy gap, were analysed. Additionally, changes in mHKA angle and total osteotomy volume were compared based on insert thickness.

**Results:**

A positive correlation was found between aHKA angle minus preoperative mHKA angle and the change in mHKA angle. The pre-osteotomy gap was also positively correlated with aHKA angle minus preoperative HKA angle. Greater changes in mHKA angle occurred in cases with inserts ≥ 9 mm compared to 8 mm inserts, though osteotomy volume did not significantly differ between the groups.

**Conclusions:**

In fixed-bearing UKA, postoperative changes in mHKA angle are likely to be more pronounced when there is a significant difference between aHKA angle and preoperative mHKA angle. Surgical planning should account for this factor to optimise outcomes.

## Introduction

Unicompartmental knee arthroplasty (UKA) has demonstrated effectiveness in treating isolated medial compartment knee osteoarthritis [1–4]. Successful UKA heavily depends on careful patient selection and proper restoration of limb alignment. Previous studies indicated that the mechanical axis was corrected by approximately 3° to 6° following medial UKA [5, 6]. Mild under-correction is generally acceptable. However, excessive residual varus alignment can increase compartment force due to medial overloading, potentially leading to failure from polyethylene wear [7–10]. Conversely, over-correction of coronal alignment can cause lateral compartment degeneration and premature prosthesis loosening [8, 9]. Therefore, a consistent method for predicting postoperative coronal alignment is needed to mitigate the risk of postoperative malalignment. According to the classical selection criteria by Kozinn et al. [11], medial UKA should only be performed in patients with a preoperative varus deformity of 15° or less, as restoring the mechanical axis to near neutral is generally not feasible in those who do not meet this criterion. Recently, these selection criteria have been re-evaluated, with reports suggesting that patients with severe deformities can achieve good function and quality of life with UKA if satisfactory mechanical alignment is attained [12]. However, the specific conditions under which this is possible remain unclear.

Recently, Nakano et al. [13] found a positive correlation between the severity of preoperative varus deformity and the pre-osteotomy medial joint gap in fixed-bearing UKA using a specially designed UKA tensor. Also, in another report by the group [14], the severity of the preoperative varus deformity as well as the intraoperative pre-osteotomy gap was found to be positively related to the amount of postoperative correction in mechanical hip-knee-ankle (HKA) angle, and thick inserts tended to be used in severe varus knees. The group also aimed to predict the post-surgery mHKA angle based on preoperative bone factors that are not influenced by joint space narrowing. They tried to clarify the relationship between a new bone measurement, the arithmetic hip-knee-ankle (aHKA) angle, and the postoperative mHKA angle after medial fixed-bearing UKA [15]. To calculate aHKA angle, lateral distal femoral angle (LDFA) and medial proximal tibial angle (MPTA) are measured separately using long leg radiographs, and an arithmetic method is subsequently used. aHKA angle is predictive of the patient’s constitutional knee alignment when comparing arthritic to non-arthritic patients [16]. It was concluded that aHKA angle was correlated with postoperative mHKA angle after medial fixed-bearing UKA. Although previous studies have shown that the amount of alignment correction by UKA increases with higher levels of preoperative varus deformation, even if the degree of varus deformity is the same, the pathology will differ depending on whether the cause is associated with the original bony morphology or with the positional relationship between the femur and tibia and the degree of cartilage wear.

This study tried to investigate whether the amount of change in mHKA angle due to medial fixed-bearing UKA can be predicted based on aHKA angle. Specifically, whether the difference between preoperative mHKA angle and aHKA angle correlated with the amount of change in mHKA angle was investigated, including factors such as intraoperative pre-osteotomy gap and insert thickness.

## Materials and methods

The hospital ethics committee at Kobe university graduate school of medicine approved the study protocol (No. 1510, Date of Approval: December 2, 2013). In this study, 101 consecutive patients (125 knees) underwent medial fixed-bearing unicompartmental knee arthroplasty (UKA) using the Persona Partial Knee System (Zimmer Biomet Inc., Warsaw, IN, USA). Informed consent was obtained from all patients, and the collected data were retrospectively analysed. The inclusion criteria for UKA were a radiographic diagnosis of isolated end-stage (Kellgren-Lawrence grade 4) medial compartment osteoarthritis (OA) or osteonecrosis (ON) with an active range of motion (ROM) of ≥ 90°, a fixed flexion deformity of ≤ 15°, and a varus deformity of ≤ 10° in mHKA angle. Patients with ON and concurrent end-stage OA were treated as having OA. Patient demographics were shown in Table 1. Magnetic resonance imaging (MRI) was performed on all cases before surgery, and those without an intact anterior cruciate ligament (ACL) or with an affected articular surface of the lateral compartment were treated with total knee arthroplasty (TKA) instead of UKA. All 125 UKA cases were included in the study, and the surgeries were performed by two senior surgeons with over 15 years of experience.

**Table 1:** Basic (Preoperative) characteristics

Variables	
Age (years)	74.17 (range, 51–89; SD: 8.0)
Gender (male: female)	30 (39 UKAs): 71 (86 UKAs)
Side of UKA (left: right)	59:66
Diagnosis (OA: ON)	106:19
aHKA angle (°)	177.2 (range, 172.3-182.4; SD: 2.1)
Preoperative HKA angle (°)	172.3 (range, 163.8-179.1; SD: 2.8)

The values are given as the mean and standard deviation for continuous variables

UKA: unicompartmental knee arthroplasty, OA: osteoarthritis, ON: osteonecrosis, HKA: hip-knee-ankle, SD: standard deviation, aHKA: arithmetic hip-knee-ankle

After inflating the tourniquet to 250 mmHg, a limited medial parapatellar approach was performed. Upon macroscopic examination, the articular surfaces of both the lateral and patellofemoral compartments were found to be intact. Minimal soft tissue release of the medial structures and osteophyte removal were then conducted. A proximal tibial osteotomy was performed using an accelerometer-based portable navigation system (OrthAlign Plus^®^, UniAlign™; OrthAlign Inc., Aliso Viejo, CA, USA), which enables precise tibial bone cuts in UKA with coronal and sagittal alignment. This system provides a measurement accuracy of ± 0.5° when measuring the angle between the OrthAlign Plus^®^ unit and the reference sensor (manufacturer’s data). For preoperative planning, the target coronal alignment of proximal tibial osteotomy was set to neutral (0°) in varus. Sagittal alignment was determined based on the original posterior tibial slope, measured with reference to the perpendicular line of the sagittal axis. The tibial sagittal axis was defined as the line connecting the anterior one-third of the medial tibial plateau and the midpoints of the tibial plafond. The sagittal alignment was set to 6.0° if the angle was ≤ 6.0°, 8.0° if the angle was ≥ 8.0°, and the target value of preoperative planning if the angle was between 6.0° and 8.0°, as in previous studies [13, 15, 17]. The target osteotomy thickness of the tibia (excluding the thickness of the bone saw blade) was set to 4 mm from the deformed region using a special stylus in all cases. Following the tibial osteotomy, a distal femoral osteotomy was performed using the spacer block technique, referring to the surface of the proximal tibial cut. Femoral rotation was carefully adjusted to align with the mechanical axis of the tibia, and the remaining osteotomies (posterior and chamfer parts) of the femur were performed. The thickness of the tibial and femoral bone cuts was measured using a calliper, and the actual osteotomies were calculated by adding the thickness of the bone saw blades (1.27 mm).

**Fig. 1:**
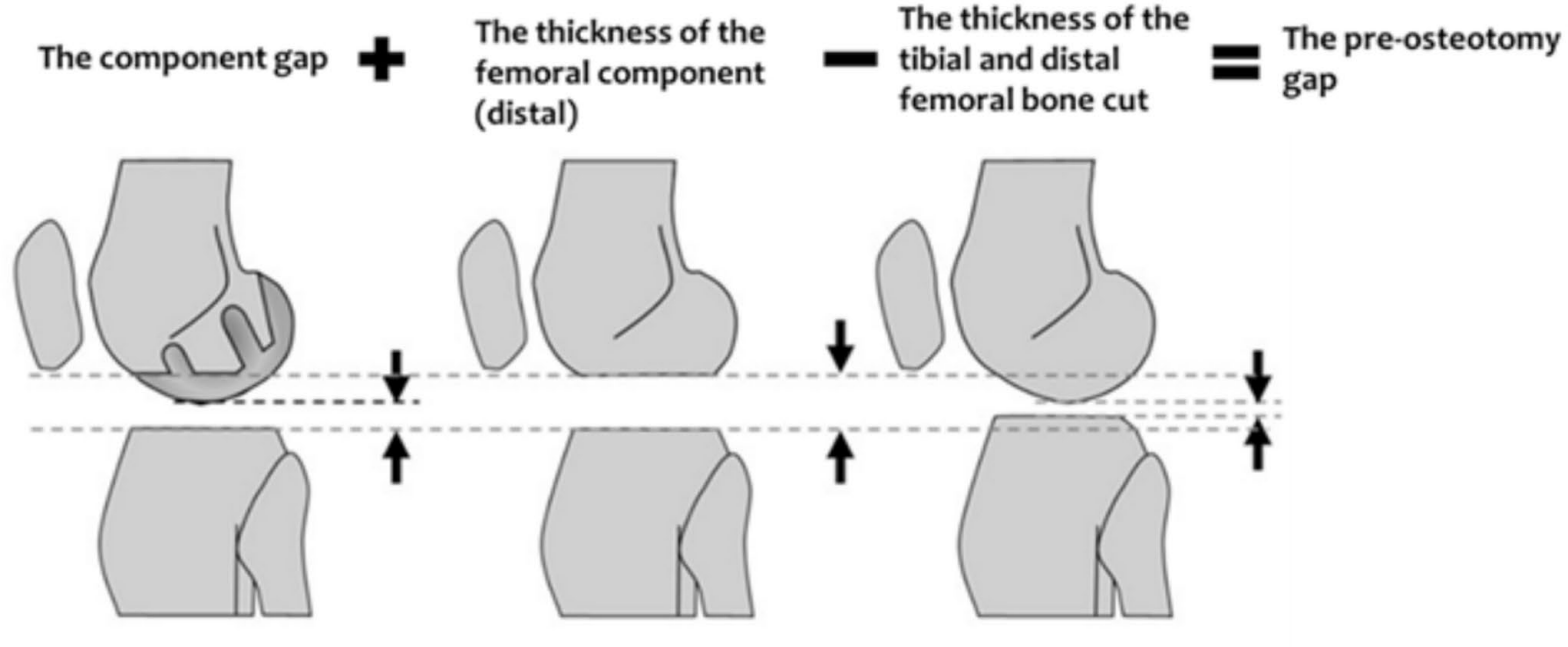
Definition of the pre-osteotomy gap. The pre-osteotomy gap = the component gap + the thickness of the femoral component - (the thickness of the tibial bone cut + the thickness of distal femoral bone cut)

**Fig. 2:**
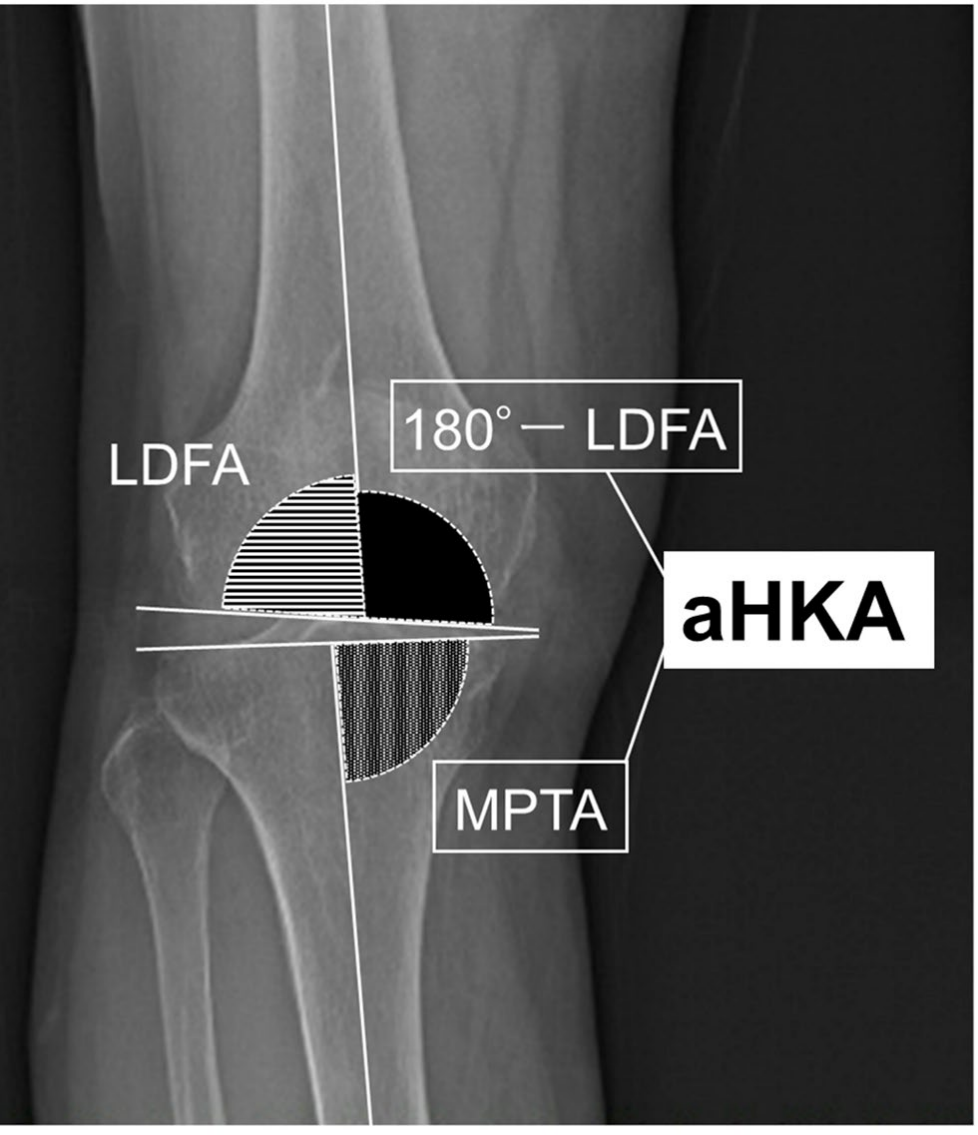
Measurement of the aHKA angle. aHKA angle was calculated as 180°– LDFA + MPTA

After performing the femoral osteotomies, a trial prosthesis was inserted into the distal femur. The gap between the medial tibial osteotomy surface and the femoral trial prosthesis was measured using a UKA tensor, a device whose design and methodology have been previously documented [18]. The UKA tensor comprises three components: an upper plate, a lower platform plate with a spike, and an extra-articular main body. The upper and lower plates are positioned in the medial compartment of the knee, allowing surgeons to measure the joint component gap under a consistent joint distraction force. In preliminary in-vitro experiments, we observed an error margin of within 3% for joint distraction. The joint distraction force was applied between the upper and lower plates using a specially designed torque driver. During measurements, the medial parapatellar arthrotomy was temporarily sutured proximal to the UKA tensor’s connection arm. To eliminate external load on the knee, the thigh and knee were aligned in the sagittal plane. The joint distraction forces were preloaded multiple times to reduce error from the surrounding soft tissues’ creep. A joint distraction force of 20 lb (9.1 kg) was applied, and the component gap (in mm) between the centre midpoints of the upper plate’s surface and the proximal tibial cut was measured in extension. The pre-osteotomy gap in extension was then calculated using the formula: pre-osteotomy gap = component gap + thickness of the femoral component - thickness of the tibial bone cut - thickness of the distal femoral bone cut (Fig. 1). Additionally, the thickness of the selected insert for each case was recorded. Pre- and postoperative coronal mHKA angles were measured using long-leg standing radiographs. LDFA was determined as the lateral angle formed by the mechanical axis of the femur and a line drawn across the articular surface of the distal femur at the lowest points of the lateral and medial femoral condyles. MPTA was measured as the medial angle between the mechanical axis of the tibia and a line connecting the most distal articular contours of the midpoints of the lateral and medial plateaus. The aHKA angle was calculated using the formula: 180°– LDFA + MPTA (Fig. 2).

The data are presented as means ± standard deviations. To assess the intra-observer and inter-observer reliability of the measurements of LDFA, MPTA, and mHKA angles, two investigators evaluated the first 10 patients twice and calculated intra-class correlation coefficients (ICCs). The ICCs for intra-observer reliability were above 0.84 (ranging from 0.84 to 1.00), and those for inter-observer reliability were above 0.82 (ranging from 0.82 to 0.96) for all measurements. Correlations between the difference of aHKA angle minus preoperative mHKA angle and the amount of change in the mHKA angle, as well as the pre-osteotomy gap, were examined using single regression analysis. Additionally, patients were categorised into two groups based on the thickness of the selected insert: Group 8 mm and Group ≥ 9 mm. Note that 8 mm is the thinnest insert and the policy is to use inserts of 9 mm or more when the medial tension is insufficient when 8 mm inserts are used. The unpaired Student’s t-test was used to compare the amount of change in the mHKA angle and the total osteotomy volume (distal femur + proximal tibia) between the two groups. Data analyses were conducted using BellCurve for Excel (Social Survey Research Information Co., Ltd., Tokyo, Japan). Sample size calculation was performed using G*Power 3 (Heinrich Heine Universität Düsseldorf, Germany). According to our calculations, a minimum sample size of 89 patients was required for the single regression analysis with an effect size f2 of 0.15, a type I error (α) of 0.05, and a power (1 − β) of 0.95. Statistical significance was defined as *P* < 0.05.

## Results

**Fig. 3:**
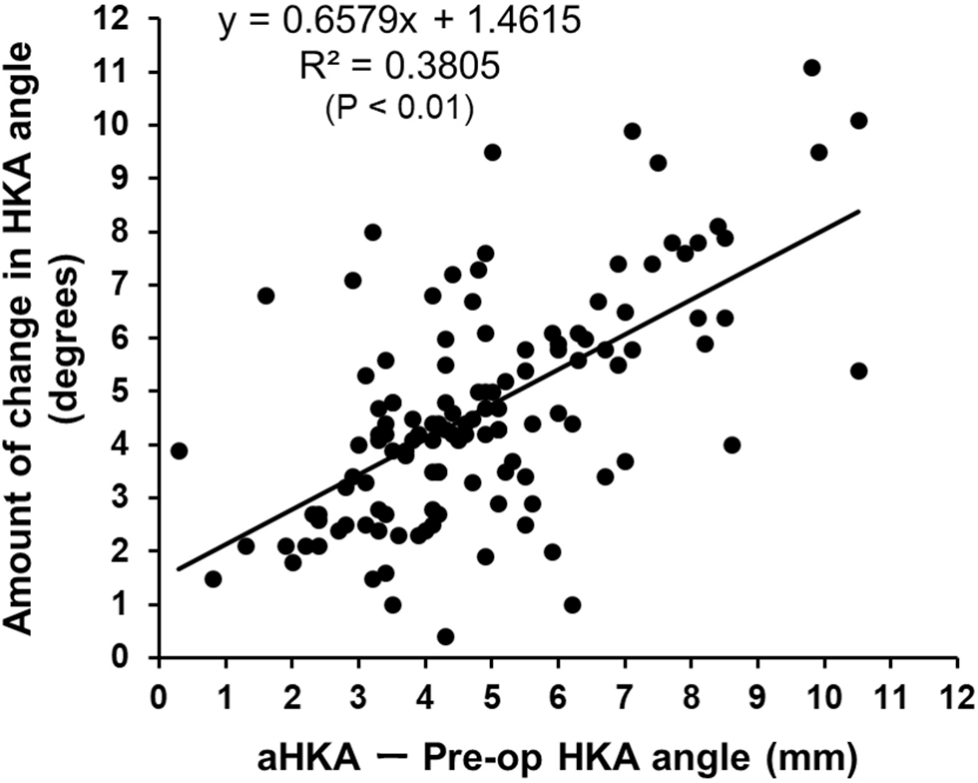
Correlation analysis between aHKA angle minus preoperative mHKA angle and the amount of change in HKA angle. A significant positive correlation was found between them (*P* < 0.01)

**Fig. 4:**
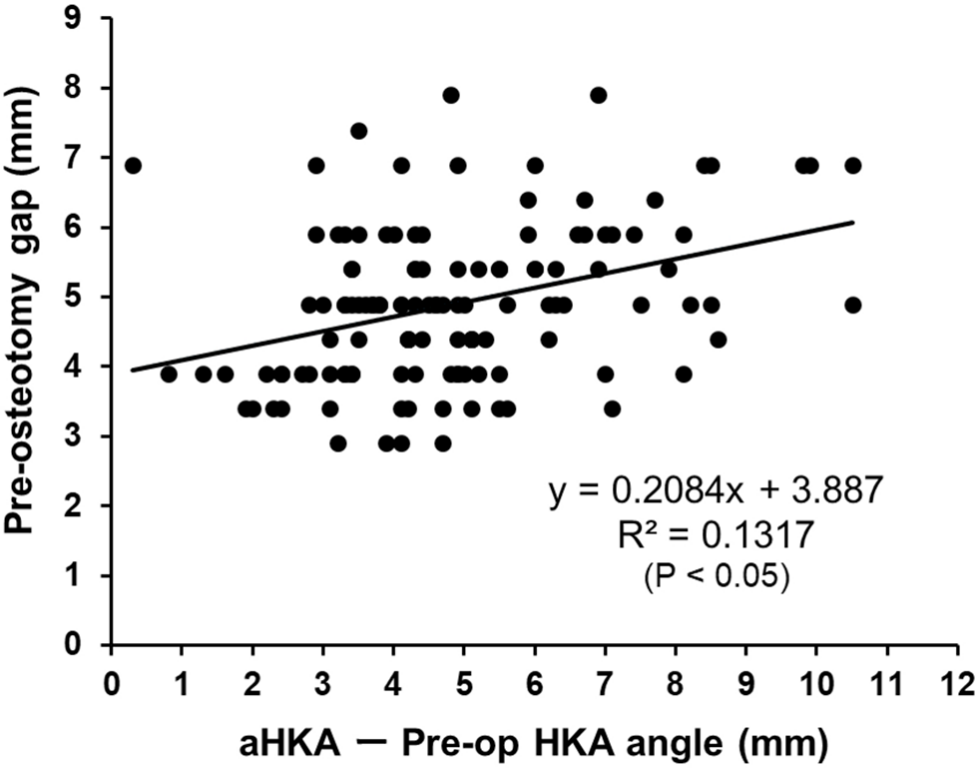
Correlation analysis between aHKA angle minus preoperative mHKA angle and the pre-osteotomy gap. A significant positive correlation was found between them (*P* < 0.05)

A total of 125 knees in 101 patients were enrolled in the study. The mean ± SD of preoperative radiological parameters, i.e., mHKA angle and aHKA angle, were presented in Table 1. The mean ± SD of intra- and postoperative variables including radiological parameters (mHKA angle and the amount of change in mHKA angle), pre-osteotomy gap, and the amount of distal femoral and proximal tibial osteotomy were shown in Table 2. Of 125 knees included in the study, 8 mm, 9 mm, 10 mm and 11 mm thick insert were used in 87, 21, 13 and 4 knees, respectively (Table 2). As for the correlation analysis between aHKA angle minus preoperative mHKA angle and the amount of change in mHKA angle, there was a positive corelation between them (R^2^ = 0.3805, *P* < 0.01) (Fig. 3). Also, pre-osteotomy gap was positively correlated with aHKA angle minus preoperative mHKA angle (R^2^ = 0.1317, *P* < 0.05) (Fig. 4).

**Table 2:** Intra- and postoperative outcomes

Variables	
Postoperative mHKA angle (°)	177.0 (range, 170.2-182.7; SD: 2.4)
Amount of change in mHKA angle (°)	4.6 (range, 0.4–11.1; SD: 2.1)
Pre-osteotomy gap (mm)	4.9 (range, 2.9–7.9; SD: 1.1)
Amount of distal femoral osteotomy (mm)	6.3 (range, 4.3–9.3; SD: 0.8)
Amount of tibial osteotomy (mm)	5.1 (range, 3.3–7.3; SD: 0.9)
Number of knees with each insert thickness	8 mm: 87, 9 mm: 21, 10 mm: 13, 11 mm: 4

The values are given as the mean and standard deviation for continuous variables

mHKA: mechanical hip-knee-ankle, SD: standard deviation

**Fig. 5:**
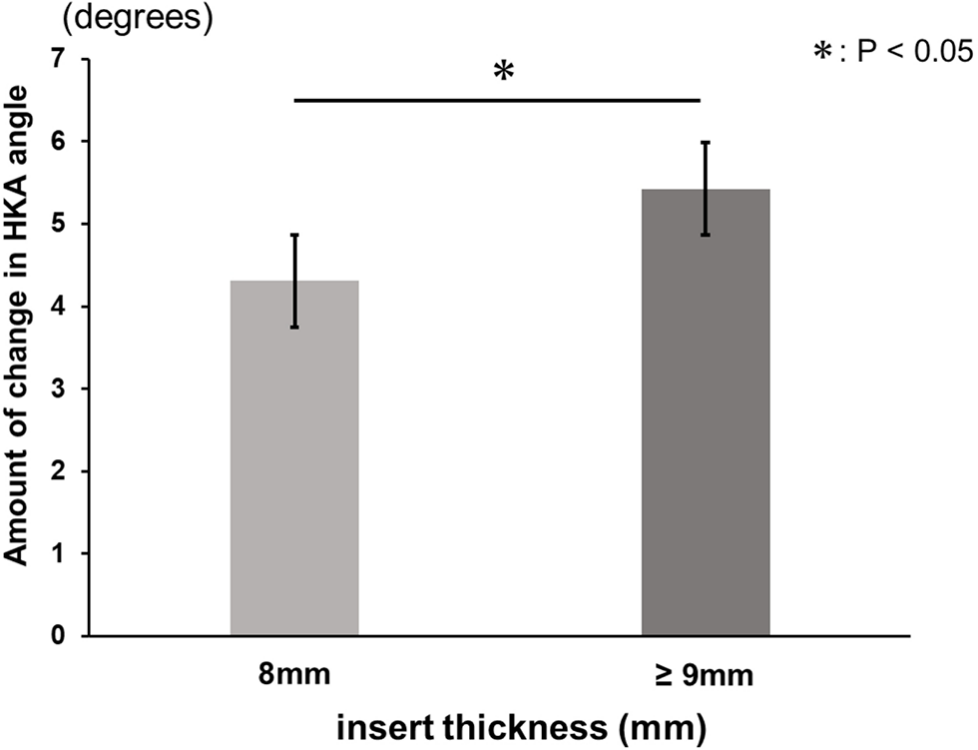
Mean and the standard deviation of the amount of change in mHKA angle for each insert thickness group (8 mm and ≥ 9 mm). Significant difference was found between the two groups (*P* < 0.05)

**Fig. 6:**
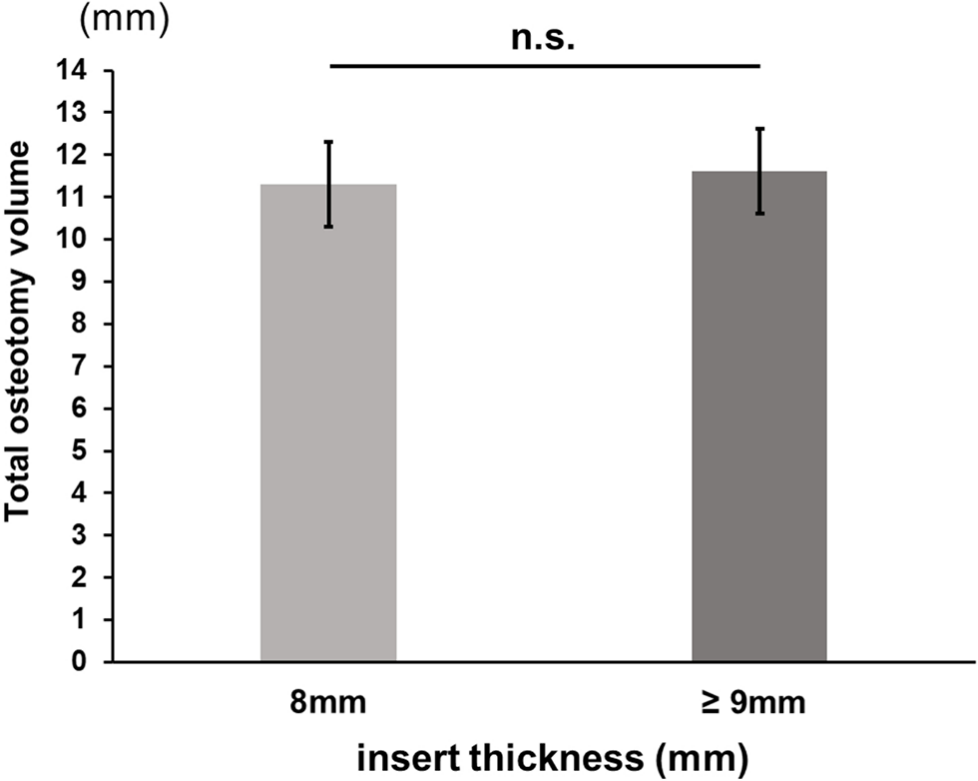
Mean and the standard deviation of the total osteotomy volume (distal femur + proximal tibia) for each insert thickness group (8 mm and ≥ 9 mm). No significant difference was found between the two groups

Comparison for change in mHKA angle between two groups according to insert thickness, the amount of change in mHKA angle was greater in Group ≥ 9 mm than in Group 8 mm (*P* < 0.05) (Fig. 5). Regarding the amount of total osteotomy, i.e., distal femoral osteotomy plus proximal tibial osteotomy, no significant difference was found between the two groups (Fig. 6).

## Discussion

The key finding of this study was that the difference between aHKA angle and preoperative mHKA angle correlated with an amount of valgus change in mHKA angle after fixed-bearing UKA using spacer block technique. Additionally, we found that the thicker insert tends to be used in the knee with more difference between aHKA angle and preoperative mHKA angle by assessing the thickness of the selected insert in each patient.

UKA is a preferred procedure due to its minimal invasiveness, excellent postoperative range of motion, and quicker recovery compared to TKA. However, despite advancements in surgical techniques and implant design, some studies indicate that UKA survival rates still do not match those of TKA [19–21]. For optimal clinical outcomes and implant longevity in UKA, precise surgical technique, particularly in alignment and soft-tissue balance, is crucial [22–24]. Aseptic loosening remains the leading cause of early postoperative failure, while the progression of lateral osteoarthritis is the most common reason for midterm and late-period failures [7]. In greater detail, undercorrection can lead to increased loading on the medial compartment, potentially accelerating polyethylene wear [25, 26]. Additionally, a significant residual varus deformity after surgery has been linked to stress fractures on the medial tibial plateau [27]. Consequently, a reliable method for predicting postoperative mHKA angle is crucial to assess the risk of coronal malalignment after surgery. However, there are only a few studies that have identified risk factors for postoperative coronal malalignment in UKA [28, 29]. To anticipate postoperative mHKA angle, some researchers suggest using a preoperative valgus stress test with radiographs, though this approach remains debated [6, 29–31]. One study found a strong correlation between changes in the mHKA angle and both the femorotibial facet angle value and its alteration on valgus stress radiographs [6]. On the other hand, another study argued that since the full extent of varus deformity correction cannot be accurately assessed until osteophytes are removed, preoperative valgus stress radiographs often lack precision [30].

In the context of fixed-bearing UKA using the spacer block technique, a positive correlation has been observed between the severity of preoperative varus deformity in the knee and the pre-osteotomy gap in extension [13]. This finding suggests that medial soft tissue tightness may not be present, even in knees with severe varus deformity. Building on this, another study found that both the severity of preoperative varus deformity and the intraoperative pre-osteotomy gap were positively associated with the degree of postoperative alignment correction. In cases of severe varus deformity, thicker inserts were more commonly used in the fixed-bearing UKA with the spacer block technique [14]. However, there is still a gap in understanding regarding the potential for varying amounts of correction with fixed-bearing UKA. Even when the preoperative mHKA angle is similar, differences in the original alignment of each case may lead to different outcomes. Recently, aHKA angle has gained attention as a method for assessing lower limb morphology, whereas the mHKA angle has traditionally been used to evaluate coronal alignment [16]. The aHKA angle method relies solely on bony landmarks and is independent of the femur-to-tibia relationship, making it unaffected by joint space narrowing. Additionally, aHKA angle is not influenced by whether the patient is standing or lying down during imaging, which is expected to reduce measurement errors between patients. A very recent study found a correlation between aHKA angle and postoperative mHKA angle following medial fixed-bearing UKA [15]. Building on these findings, the present study demonstrated that aHKA angle can be effectively used to predict the extent of change in mHKA angle resulting from medial fixed-bearing UKA. Compared to previous studies [6, 14, 29–32], this method offers greater reproducibility, as it relies on aHKA angle, which is not influenced by soft tissue balance.

The spacer block method played a crucial role in achieving the current results. This technique typically involves resecting a fixed amount of the proximal tibia, usually around 4 mm, followed by aligning the distal femoral resection parallel to the tibial resection in extension, once all osteophytes have been removed. The amount of osteotomy performed on the distal femur is carefully matched to the thickness of the distal part of the femoral implant. Finally, the thickness of the insert is determined by assessing the tension between the femur and tibia using a specialised spatula. This evaluation method was consistently applied across all cases, regardless of the severity of the varus deformity. Because the osteotomy amounts in the distal femur and proximal tibia are fixed, the gap between the distal femoral component and the proximal tibia tends to be wider in knees where there is a greater difference between aHKA angle and the preoperative mHKA angle. This can result in a more pronounced valgus change in the mHKA angle. The insert thickness also plays a critical role in this process. Since the osteotomy amounts in the distal femur and proximal tibia are usually the same, a larger gap is created in knees with a greater discrepancy between the aHKA angle and preoperative mHKA angle. As a result, these knees require thicker inserts to achieve the proper tension between the distal femur and proximal tibia.

Based on this, it may be beneficial to reduce the amount of osteotomy in such cases. One advantage of reducing proximal tibial osteotomy is the potential for decreased pain due to reduced medial stress, as this approach enhances medial support. The strength of the proximal tibial bone diminishes as the distance from the subchondral resection surface increases [33], supporting the theory that a more extensive tibial bone cut may result in a weaker bone bed for the tibial component. Additionally, it has been reported that excessive tibial resection can increase the forces on the tibial surface, potentially leading to pain [34, 35]. Another benefit of reducing the tibial bone cut is the ability to use a larger tibial implant size. With less bone removed, the remaining proximal tibial bone, which has an inverted cone shape, can support a larger tibial implant. Excessive bone cutting can result in a smaller tibial bone bed, potentially leading to collapse or loosening of the tibial plateau [36, 37]. This is particularly advantageous for patients with smaller body sizes, as it allows for the use of available tibial prosthesis sizes that might otherwise be unsuitable.

This study has several limitations. Firstly, it relied solely on plain radiographs, which may lead to inaccuracies in measuring lower extremity alignment. A CT-based approach might offer more precise evaluations of alignment. Secondly, the study did not assess clinical outcomes, and thus, the impact of changes in mHKA angle following UKA on clinical outcomes, including patient-reported outcome measures, remains unclear. Long-term follow-up is needed to explore this relationship further. Thirdly, radiographic measurements such as pre- and postoperative mHKA angle, LDFA, and MPTA could be influenced by lower extremity rotation and osteophyte formation, though intra-observer and inter-observer agreements for these measurements were within acceptable limits. Lastly, this study was a retrospective analysis focusing on fixed-bearing medial UKA with the spacer block technique. Therefore, the applicability of these results to mobile-bearing medial UKA or fixed-bearing medial UKA using other surgical techniques, such as the intramedullary rod method, remains uncertain.

## Conclusions

In medial fixed-bearing UKA with the spacer block technique, postoperative changes in mHKA angle are likely to be more significant when there is a substantial difference between aHKA angle and preoperative mHKA angle. Thick inserts are also more likely to be used in such cases, and consideration may be given to reducing the amount of tibial osteotomy. Surgical planning should consider these factors to optimise outcomes.

## Data Availability

No datasets were generated or analysed during the current study.
